# SNPTrack^TM^ : an integrated bioinformatics system for genetic association studies

**DOI:** 10.1186/1479-7364-6-5

**Published:** 2012-07-05

**Authors:** Joshua Xu, Reagan Kelly, Guangxu Zhou, Steven A Turner, Don Ding, Stephen C Harris, Huixiao Hong, Hong Fang, Weida Tong

**Affiliations:** 1 ICF International at NCTR, 3900 NCTR Rd, Jefferson, AR, 72079, USA; 2 Division of Bioinformatics and Biostatistics, National Center for Toxicological Research, US Food and Drug Administration, 3900 NCTR Rd, Jefferson, AR, 72079, USA

## Abstract

A genetic association study is a complicated process that involves collecting phenotypic data, generating genotypic data, analyzing associations between genotypic and phenotypic data, and interpreting genetic biomarkers identified. SNPTrack is an integrated bioinformatics system developed by the US Food and Drug Administration (FDA) to support the review and analysis of pharmacogenetics data resulting from FDA research or submitted by sponsors. The system integrates data management, analysis, and interpretation in a single platform for genetic association studies. Specifically, it stores genotyping data and single-nucleotide polymorphism (SNP) annotations along with study design data in an Oracle database. It also integrates popular genetic analysis tools, such as PLINK and Haploview. SNPTrack provides genetic analysis capabilities and captures analysis results in its database as SNP lists that can be cross-linked for biological interpretation to gene/protein annotations, Gene Ontology, and pathway analysis data. With SNPTrack, users can do the entire stream of bioinformatics jobs for genetic association studies. SNPTrack is freely available to the public at http://www.fda.gov/ScienceResearch/BioinformaticsTools/SNPTrack/default.htm.

## Introduction

Personalized medicine will improve health outcomes and patient satisfaction. However, implementing personalized medicine based on individuals' biological information relies on genetic biomarkers that are identified through genetic association studies. High-throughput genotyping technologies have been advanced to enable the simultaneous determination of genotypes for millions of single-nucleotide polymorphisms (SNPs). Concurrently, the International HapMap Project determined genotypes of over 3.1 million common SNPs in human populations [1]. These advances combine to make genetic association studies a feasible and promising research field for personalized medicine. However, there are a number of bioinformatics challenges associated with the enormous amount of genetic data generated by high-throughput technologies. Storing and accessing the data, performing association tests, and interpreting results can no longer be readily done using *ad hoc* approaches commonly utilized for much smaller candidate gene association studies. Furthermore, because contributions of individual polymorphisms to a phenotype are typically quite small, appropriate analysis and interpretation techniques are key. Thus, identifying all associated polymorphisms and placing them in context is a necessary step in understanding their role in defining the phenotype or treatment response.

A number of bioinformatics algorithms and tools have been developed for managing and analyzing genetic data as well as for interpreting genetic biomarkers. However, none of them have been able to do all of the bioinformatics jobs needed for a complete genetic association study; scientists have needed to use more than one tool for their studies. Therefore, there was high demand for an integrated bioinformatics system.

Early in the Voluntary eXploratory Data Submission program [2], the FDA's National Center for Toxicological Research developed ArrayTrack^TM^ to manage, analyze, and interpret microarray gene expression data [3,4]. ArrayTrack^TM^ has since been used for reviewing and analyzing genomic data at the FDA and for genomic research in the scientific community. Building on the success and experience from ArrayTrack^TM^, SNPTrack was developed as a one-stop-shop bioinformatics solution capable of performing the same function for genetic data that ArrayTrack^TM^ does for gene expression data. SNPTrack offers a full suite of data storage and management, analysis, and interpretation tools for genetic association studies.

## Implementation

**Figure 1:**
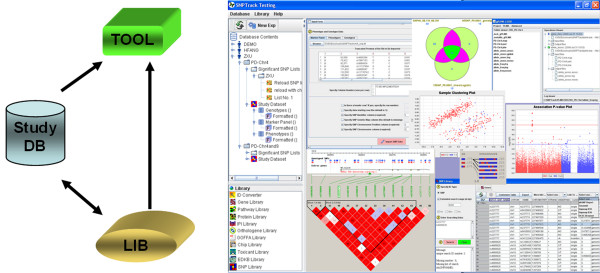
SNPTrack's graphical user interface with the connections of its major components: StudyDB, TOOL, and LIB.

SNPTrack adopts a client‐server system that integrates data management, analysis, and interpretation into a single system. The Oracle server stores and integrates phenotypic and genotypic data as well as annotations of genetic biomarkers from public resources about SNPs, quantitative trait loci (QTLs), genes, proteins, and pathways. Its user interface, query mechanism, and data visualization features were implemented in Java. As depicted in Figure 1, SNPTrack has three major components: StudyDB, TOOL, and LIB.

StudyDB hosts and manages genotypic and phenotypic data. It supports importing of three types of files in tab-delimited text format: annotation files for the genotyped SNPs (which is compiled for the study or provided by the chip provider), genotype data files, and phenotype data files (which may include sex, age, race, disease status, and drug information such as environmental exposure, dose, treatment response, and adverse events). Data are organized and presented in a tree-structured view of three node types: study owner or group (username), study title, and study data.

The TOOL component provides the data analysis features. Data are formatted and exported to the client computer for analysis with PLINK, a command-line program that features many statistical methods such as case‐control associations, various regression methods, permutation tests, false discovery rate, and other algorithms [5]. Analysis commands in PLINK are issued and managed through gPLINK, a Java-based graphical user interface for PLINK commands management [6]. Analysis results can be visualized through Haploview [7]. Linkage disequilibrium and haplotypes in the region around an interesting SNP can be downloaded from HapMap and viewed in Haploview. These component tools are automatically loaded to the client computer and updated by SNPTrack. Interesting SNPs can also be saved into StudyDB. As needed, other stand-alone analysis tools such as SAS and R/Bioconductor can be integrated in the TOOL.

The LIB contains a collection of libraries to facilitate the interpretation of results from genetic studies. The libraries partially mirror the contents of dbSNP, GenBank, SWISS-PROT, LocusLink, Kyoto Encyclopedia of Genes and Genomes, Gene Ontology (GO), and others. The annotations from these databases are extracted to construct the enriched libraries, such as the SNPLib, GeneLib, ProteinLib, and PathwayLib. The SNP and QTL libraries are specifically designed for genetic association studies [8]. The libraries are cross-linked and support functions such as list-based queries to provide a mechanism for data interpretation. The SNP Library follows the release cycles of dbSNP and is updated about twice a year.

A typical workflow begins with importing the SNP panel, genotype, and phenotype data files into SNPTrack. Access permission (data security) is controlled by the user. Significantly associated SNPs can be identified using PLINK. Some commonly used operations include filtering SNPs using the Hardy-Weinberg test for linkage disequilibrium, followed by an allele frequency summary, allelic association tests, genotypic association tests, and/or linear/logistic regression analysis. Significantly associated SNPs found by the analysis tools can be saved as a SNP list in SNPTrack. Users can also import, export, edit, manage, and compare SNP lists. Specific interesting SNPs can be directly linked to a wide selection of external databases (dbSNP Report, Ensembl, Hapmap, etc.) for more detailed information. Integrated libraries allow users to find genes and pathways related to SNPs.

## Availability

The SNPTrack client application works on all major operating systems including Windows, Linux, and Mac. An instance of the SNPTrack server is hosted by the FDA and freely available at http://www.fda.gov/ScienceResearch/BioinformaticsTools/SNPTrack/default.htm. Users may also request the software for a local installation. Manuals and sample data are available at the above website.

## Conclusions

SNPTrack is a one-stop-shop system for managing, analyzing, and interpreting genetic association data. It provides a centralized storage solution that can perform complicated genetic association analyses on a large number of SNPs for identification of genetic biomarkers, and find related genes, pathways, and GO terms. SNPTrack is used not only for review and analysis of genetic data by the FDA, but is also freely available to the public.

## Endnote

The views presented in this article do not necessarily reflect those of the Food and Drug Administration.

## Competing interests

The authors declare that they have no competing interests.

## Authors' contributions

JX and GZ developed SNPTrack. WT and HF conceived the original idea and methods; JX and SH guided the development. ST, SH, and JX contributed to the construction of SNPTrack databases. HF, DD, RK, and HH contributed to testing and improving the software. JX, RK, DD, and HF wrote the first draft. HH and WT improved the manuscript. All authors read and approved the final manuscript.
